# Sensory nerves regulate bone homeostasis in response to mechanical unloading or reloading: evidence from a spaceflight experiment and ground-based mechanical unloading models

**DOI:** 10.1038/s41413-026-00563-z

**Published:** 2026-08-28

**Authors:** Shohei Tsujino, Hiroki Ochi, Risa Okada, Daisuke Kamimura, Kurando Utagawa, Takaei Shin, Hironori Yamada, Aiko Unno, Satoko Sunamura, Chihiro Akazawa, Masafumi Muratani, Dai Shiba, Toshitaka Yoshii, Shingo Sato

**Affiliations:** 1 Department of Orthopaedic Surgery, Institute of Science Tokyo, Bunkyo-ku, Tokyo Japan; 2 Department of Rehabilitation for Motor Functions, Research Institute, National Rehabilitation Center for Persons with Disabilities, Tokorozawa, Saitama Japan; 3 Space Environment Utilization Center, Human Spaceflight Technology Directorate, JAXA, Tsukuba, Ibaraki Japan; 4 Department of Orthopaedics, Vanderbilt University Medical Center, Nashville, TN USA; 5 Department of Medical Chemistry, Graduate School of Medicine, Kyoto University, Kyoto, Japan; 6 Department of General Medicine, Institute of Science Tokyo, Bunkyo-ku, Tokyo Japan; 7 Kawasaki Municipal Tama Hospital, Tama-ku, Kanagawa Japan; 8 Intractable Disease Research Center, Juntendo University Graduate School of Medicine, Bunkyo-ku, Tokyo Japan; 9 Department of Genome Biology, Transborder Medical Research Center, Institute of Medicine, University of Tsukuba, Tsukuba, Ibaraki Japan; 10 Center for Innovative Cancer Treatment, Institute of Science Tokyo, Bunkyo-ku, Tokyo Japan

**Keywords:** Bone, Homeostasis

## Abstract

Mechanical loading is crucial for maintaining bone mass, and understanding how bone remodels in response to mechanical loading is essential for treating musculoskeletal disorders. Recently, sensory nerves inside bone were identified as mechanical regulators of bone homeostasis. However, how the structure and networks of sensory nerves are altered in response to changes in mechanical loading remains unclear. To address this question, we performed a spaceflight experiment on the International Space Station (ISS) and used two ground-based mechanical unloading models, tail suspension (TS) and hindlimb immobilization. In a spaceflight experiment, *Sox10-Venus* mice, in which green fluorescent protein is expressed in nerve fibers, were housed at the ISS for 3 weeks, and we demonstrated that microgravity exposure significantly decreased bone mass and impaired nerve abundance inside the bone. These changes were reproduced in both ground-based models. Mechanical reloading after TS induced the recovery of bone mass and nerve abundance. Retrograde tracing experiments revealed that mechanical changes specifically affected sensory innervation inside bone without altering the number of neurons in the dorsal root ganglia. Notably, continuous administration of calcitonin gene-related peptide (CGRP), a neuropeptide secreted from sensory nerves, prevented unloading-induced bone loss. Furthermore, both surgical and genetic ablation of sensory nerves inhibited loading-induced trabecular bone recovery. These findings demonstrate that sensory nerves inside bone play a critical role in regulating bone homeostasis in response to mechanical changes and may aid in the development of therapeutic strategies for bone loss induced by mechanical unloading, such as prolonged bed rest and long-term space travel.

## Introduction

As their prevalence is increasing, osteoporosis and fragility fractures represent critical medical problems in our aging society.^1^ Reduced physical activity and prolonged bed rest, which are common among elderly individuals, lead to mechanical unloading of bone, resulting in accelerated bone loss and increased fracture risk.^2^ Moreover, treatments for osteoporosis and fragility fractures impose significant medical and socioeconomic burdens.^3,4^ Understanding the mechanisms of age-related musculoskeletal disorders and how mechanical loading maintains bone homeostasis is crucial for developing novel strategies to address these conditions and increase healthy life expectancy.

Bone is a dynamic tissue that is continuously remodeled in response to mechanical loading. While osteocytes are traditionally considered the primary mechanosensors in bone,^5–7^ recent research has revealed that the somatosensory system, which is composed of peripheral nerves, also plays a crucial role in bone homeostasis.^8–10^ Sensory nerves inside bone express specific neuropeptides, such as calcitonin gene-related peptide (CGRP) and substance P (SP),^11–13^ and the majority (~77%) of these sensory nerves are CGRP positive.^14^ The neuropeptides expressed by these nerves interact with corresponding receptors on bone cells to regulate bone metabolism. Our group demonstrated that sensory innervation is essential for maintaining bone mass through studies of Sema3A-deficient mice^15^ and found that disrupted innervation impairs bone formation via nerve ablation studies.^16^ Moreover, tropomyosin receptor kinase A (TrkA), which binds nerve growth factor (NGF), is highly expressed in sensory nerves,^17^ and several groups have shown that sensory nerves regulate bone formation and control biomechanical responses through NGF/TrkA signaling.^18,19^ On the basis of these studies, sensory nerves inside bone are considered essential regulators of the mechanical response and bone metabolism. However, the precise mechanisms by which the structure and networks of sensory nerves are altered in response to changes in loading conditions and their effects on bone metabolism remain unclear.

Recently, we reported a novel bone clearing technique, the Osteo-DISCO method, which enables three-dimensional visualization of nerves inside bone.^16^ We made further advancements in imaging technology to obtain higher-resolution images by using different types of microscopes to enable more detailed observation of nerve distribution (Fig. S1a, Supplementary Movies 1, 2). This increase in resolution allowed us to perform a quantitative assessment of the amount of nerve fibers inside bone (Fig. S1b).

Spaceflight experiments offer a unique opportunity to research the biological functions of bone under microgravity conditions. Such experiments provide the most accurate conditions for conducting studies on the physiological effects of mechanical unloading and overcome the limitations of ground-based approaches such as incomplete mechanical unloading and the potential influences of physical restraints. Moreover, since the space environment accelerates the aging process, with bone loss occurring approximately 10 times faster and muscle loss occurring two times faster in space than on Earth,^20–24^ spaceflight experiments can be used to elucidate the mechanisms of osteoporosis in elderly individuals on Earth. Therefore, the use of spaceflight experiments is considered a critical approach for advancing our understanding of age-related bone disorders.

In the present study, the role of sensory nerves inside bone was comprehensively investigated through multiple experimental approaches, including a spaceflight experiment on the International Space Station (ISS) and sensory nerve manipulations such as CGRP administration and surgical or genetic nerve ablation.

## Results

### Microgravity exposure in space induces bone loss and decreases nerve abundance inside bone

**Fig. 1:**
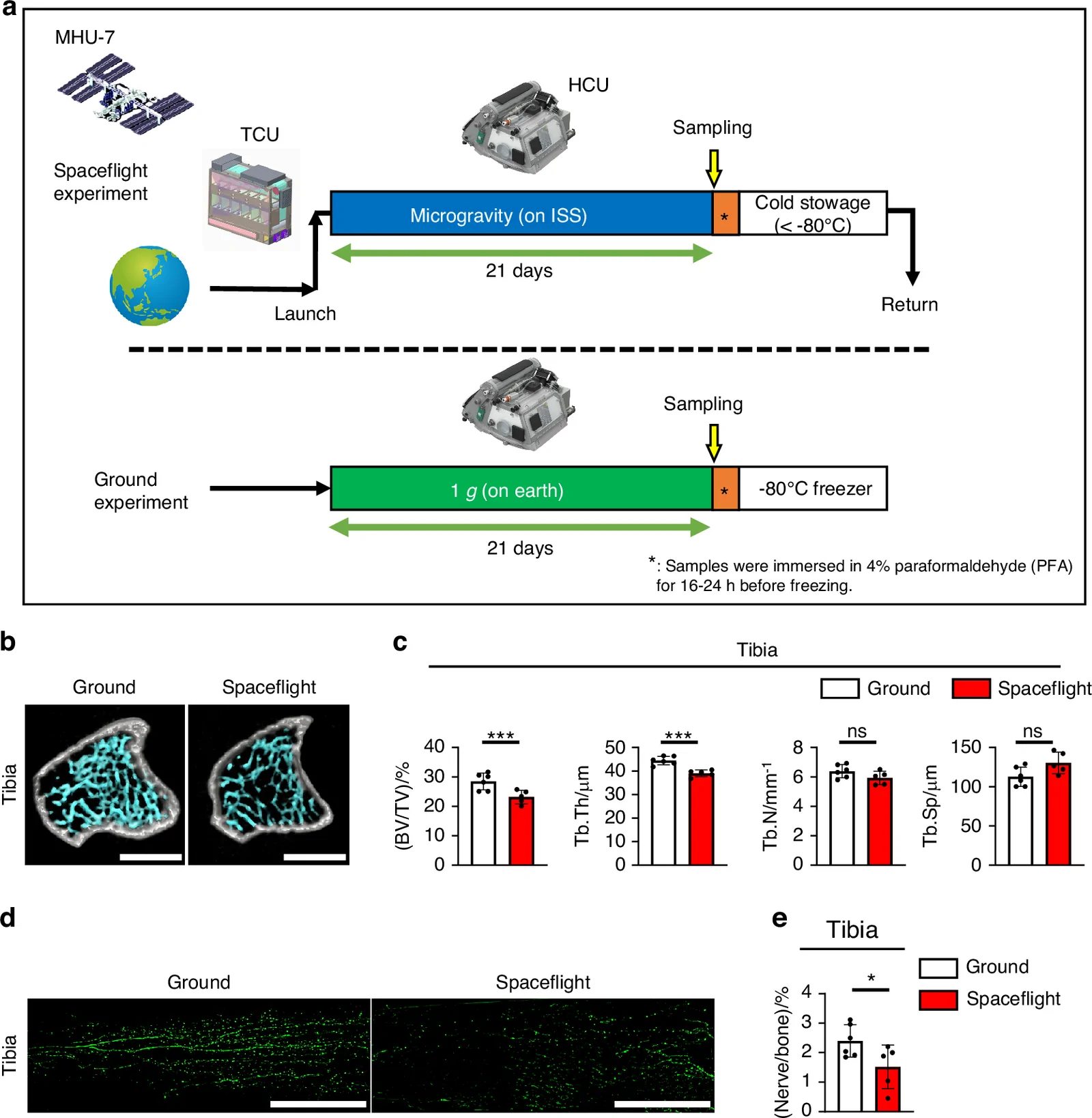
Microgravity exposure in space induces bone loss and decreases nerve abundance inside bone. **a** Flow chart of the procedure from launch through the return of processed tissues in the Mouse Habitat Unit 7 (MHU-7) mission. Six 11-week-old male *Sox10-Venus* mice were placed into a transportation cage unit (TCU) one day before launch. After they reached the International Space Station (ISS), they were transferred to habitat cage units (HCUs). Six age-matched male mice (in the control group) were housed in HCUs on the ground. After 21 days, the right limbs were collected, fixed in 4% paraformaldehyde (PFA) for 16–24 h, and then frozen for further analyses. **b** Representative microcomputed tomography (micro-CT) images of the proximal tibiae of *Sox10-Venus* mice housed on the ground or in space. Scale bar, 1 mm. **c** Quantitative analyses of bone volume and microstructural indices of trabecular bone in proximal tibiae. BV/TV bone volume/total volume, Tb.Th trabecular thickness, Tb.N trabecular number, Tb.Sp: trabecular separation (*n* = 6 on the ground and *n* = 5 in space). **d** Representative microscopy images of nerves inside the proximal tibiae of mice housed on the ground and in space. Scale bar, 1 mm. **e** Quantitative analysis of the proportion of nerve fibers in the bone area (% nerve/bone ratio) in proximal tibiae (*n* = 6 on the ground and *n* = 5 in space). The data are presented as the means ± standard deviations (SDs). Two-tailed unpaired Student’s *t* tests were used for statistical analysis. *P < 0.05; ***P < 0.001; ns indicates not significant

To elucidate the impact of microgravity on neural and vascular networks in musculoskeletal tissue, we conducted a series of experiments on the ISS. Six male *Sox10-Venus* mice, in which green fluorescent protein is expressed in nerve fibers, were launched to the ISS at 11 weeks of age and housed for 3 weeks in the Japanese Experiment Module Kibo using JAXA’s mouse habitat cage units (HCUs) (Fig. 1a). The health status of each mouse was monitored daily by veterinarians on the ground through a downlinked video system, and no notable differences between the mice and the 6 control mice housed on the ground at Kennedy Space Center were detected, except in terms of gravitational load.

Previous studies have shown that exposure to microgravity reduces bone and muscle mass.^25–28^ To verify that this was reproduced under our experimental conditions, we first examined changes in bone and muscle. Microcomputed tomography (micro-CT) analysis of the proximal tibiae and distal femurs revealed a significant decrease in trabecular bone mass in the mice subjected to spaceflight, as determined by a reduced bone volume/tissue volume (BV/TV) and trabecular bone thickness (Tb.Th) (Fig. 1b, c and Fig. S2a, b). In the cortical bone analysis, no significant changes were observed in the tibiae, whereas the femurs showed significant decreases in cortical thickness (Ct.Th) and BMD in the spaceflight group (Fig. S2c). With respect to muscle mass, different changes in weight were observed for different muscles in the mice subjected to spaceflight. Significant muscle weight loss was observed in antigravity muscles, such as the soleus and plantaris, and the weight loss of the gastrocnemius (*P* = 0.09) and quadriceps (*P* = 0.08) muscles was almost statistically significant. In contrast, fast-twitch muscles, such as the tibialis anterior and extensor digitorum longus, showed no changes in weight (Fig. S2d). These findings are consistent with those of previous spaceflight studies,^29,30^ which revealed the preferential effects of microgravity exposure on antigravity muscles during spaceflight.

Since bones are densely innervated by both neural and vascular networks,^11,17,31^ we next analyzed changes in the bone networks. To evaluate nerve abundance inside the bone, we visualized the nerves inside the tibia using the Osteo-DISCO method and quantified the amount of nerve fibers using captured images. Interestingly, the Venus fluorescence signals were significantly lower in the mice subjected to spaceflight than in the mice housed on the ground (Fig. 1d, e). To assess the vascular network inside the bone, immunofluorescence (IF) staining of the femur was performed using anti-endomucin (EMCN) and anti-VE-cadherin antibodies. The volume of EMCN-positive vessels and VE-cadherin-positive vessels in the femur remained unchanged after 3 weeks of exposure to space (Fig. S2e, f).

These findings from the spaceflight experiment demonstrate that exposure to microgravity not only decreases bone mass but also decreases the nerve abundance inside bone.

### Tail suspension (TS) and hindlimb immobilization (HLI) cause changes similar to those observed in the spaceflight experiment

**Fig. 2:**
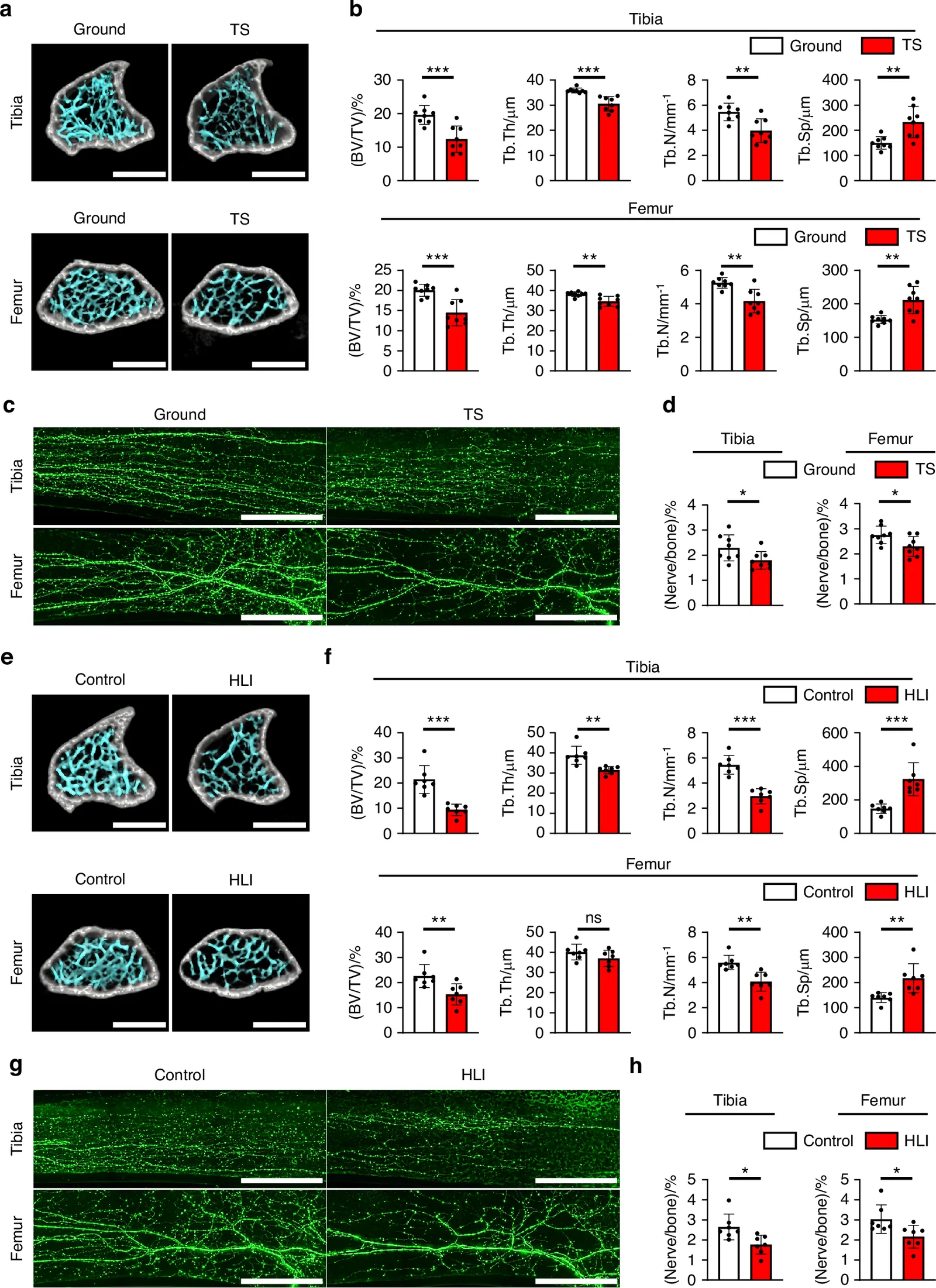
Tail suspension (TS) and hindlimb immobilization (HLI) cause changes similar to those observed in the spaceflight experiment. **a** Representative micro-CT images of the proximal tibiae and distal femurs of *Sox10-Venus* mice subjected to TS. Scale bar, 1 mm. **b** Quantitative analyses of bone volume and microstructural indices of trabecular bone in the proximal tibiae and distal femurs of mice subjected to TS (*n* = 8 per group). **c** Representative microscopy images of nerves inside the proximal tibiae and femurs of mice subjected to TS. Scale bar, 1 mm. **d** Quantitative analysis of the % nerve/bone ratio in the proximal tibiae and femurs of mice subjected to TS (*n* = 8 per group). **e** Representative micro-CT images of the proximal tibiae and distal femurs of *Sox10-Venus* mice subjected to HLI. Scale bar, 1 mm. **f** Quantitative analyses of bone volume and microstructural indices of trabecular bone in the proximal tibiae and distal femurs of mice subjected to HLI (*n* = 7 per group). **g** Representative microscopy images of nerves inside the proximal tibiae and femurs of mice subjected to HLI. Scale bar, 1 mm. **h** Quantitative analysis of the nerve/bone ratio(%) in the proximal femurs of mice subjected to HLI (*n* = 7 per group). The data are presented as the means ± SDs. Two-tailed unpaired Student’s *t* tests were used for statistical analysis. *P < 0.05; **P < 0.01; ***P < 0.001; ns indicates not significant

To validate the microgravity-induced alterations reported above in a terrestrial setting, a TS model and an HLI model were used. First, age-matched 11-week-old male *Sox10-venus* mice were subjected to TS for 3 weeks to limit ground reaction forces and mimic the effects of microgravity on the hind limbs. Micro-CT analysis revealed significant decreases in the trabecular bone mass and cortical bone volume in both the tibiae and femurs of the mice subjected to TS (Fig. 2a, b and Fig. S3a, b). Furthermore, the fluorescence signals of Venus, which labeled nerve fibers inside the bones, were significantly decreased in both the tibiae and the femurs (Fig. 2c, d).

Next, mice of the same age and sex were subjected to HLI for 3 weeks. Consistent with what was observed for the TS model, micro-CT analysis revealed decreased trabecular bone mass in both the tibiae and femurs of the mice subjected to HLI (Fig. 2e, f), accompanied by a reduced amount of nerve fibers inside the bones (Fig. 2g, h). In the cortical bone analysis, the Ct.Ar/Tt.Ar ratio was also decreased in both the tibiae and femurs (Fig. S3c, d). These ground-based experiments demonstrate that mechanical unloading induces decreases in both bone mass and nerve abundance.

The consistent changes observed across all three experimental conditions, i.e., spaceflight, TS, and HLI, raise the possibility that nerves inside bone act as key regulators to maintain trabecular bone mass in response to mechanical loading.

### Mechanical unloading affects sensory innervation inside bone

Sample et al. and Mei et al. reported that sensory nerve signaling mediates bone formation in response to mechanical loading.^32,33^ However, the detailed changes in sensory nerves under mechanical unloading remain unclear. Therefore, we performed retrograde tracing experiments, focusing particularly on TrkA-positive and CGRP-positive sensory nerves, which are known to be important regulators of skeletal homeostasis and adaptation.^14,16,18,19^

**Fig. 3:**
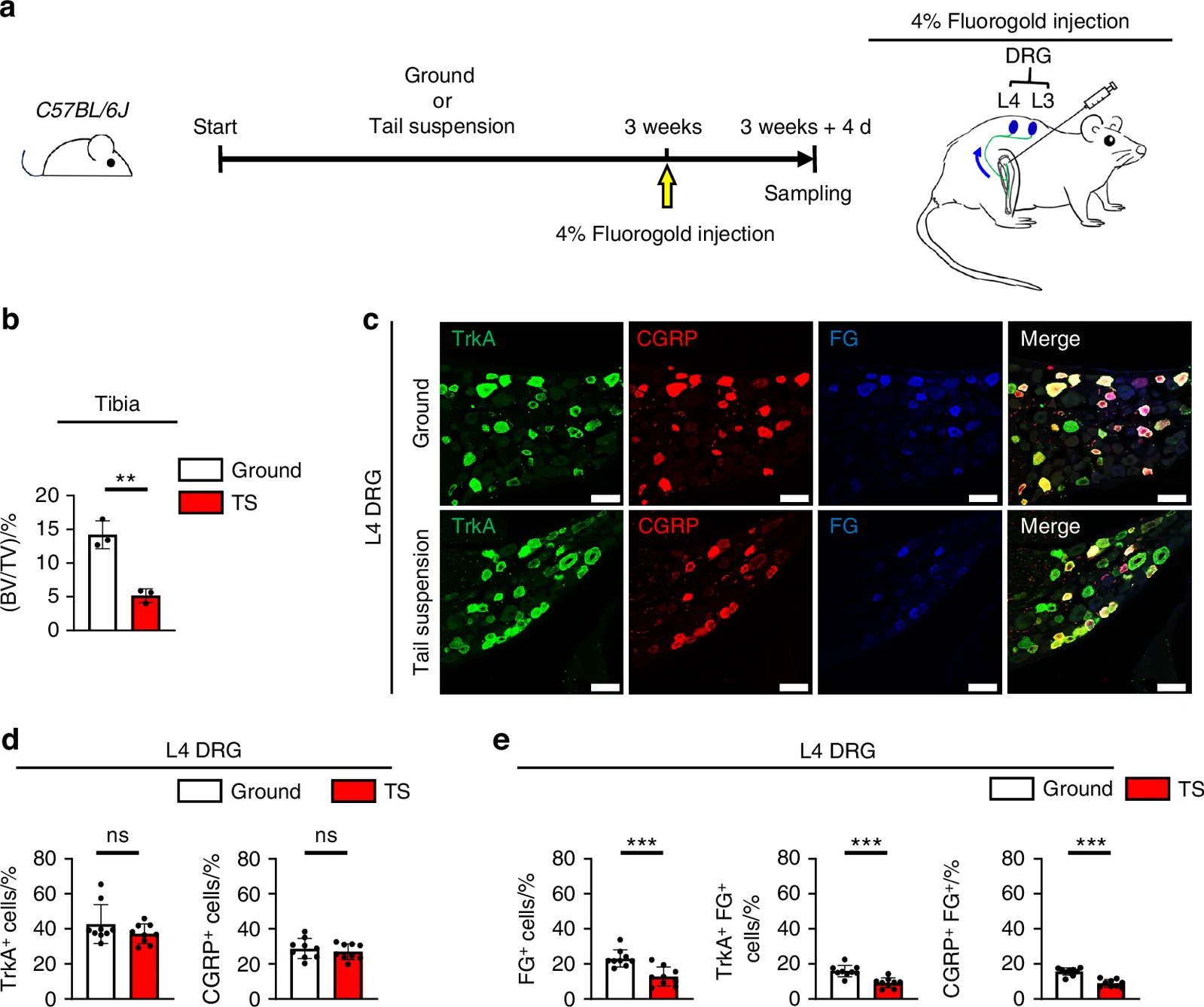
Mechanical unloading affects sensory innervation inside bone. **a** Experimental scheme of retrograde tracing. Two microliters of 4% fluorogold (FG) was injected into the right proximal tibiae of *C57BL/6J* mice after 3 weeks of TS. **b** Quantitative analysis of the bone volume/tissue volume (BV/TV) of the proximal tibia (*n* = 3 per group). **c** Representative immunofluorescence (IF) images of the L4 dorsal root ganglion (DRG). Scale bar, 50 µm. TrkA: tropomyosin receptor kinase A. CGRP: calcitonin gene-related peptide, FG: fluorogold. **d** Quantitative analysis of the percentage of CGRP- and TrkA-positive cells in the L4 DRG (*n* = 3; 9 sections per group). **e** Quantitative analysis of the percentage of FG-positive cells and cells coexpressing FG and either CGRP or TrkA in the L4 DRG (*n* = 3; 9 sections per group). The data are presented as the means ± SDs. Two-tailed unpaired Student’s *t* tests were used for statistical analysis. **P < 0.01; ***P < 0.001; ns indicates not significant

Male *C57BL/6J* mice underwent TS for 3 weeks, followed by the injection of 2 µL of 4% fluorogold (FG) into the right proximal tibia. Samples were collected 4 days post-injection (Fig. 3a). We confirmed a decrease in trabecular bone mass in the proximal tibia after TS using micro-CT (Fig. 3b). Immunohistochemical analysis of the L3 and L4 dorsal root ganglia (DRGs) revealed that the percentages of TrkA-positive and CGRP-positive cells remained unchanged (Fig. 3c, d and Fig. S4a). However, the percentage of FG-positive cells significantly decreased in mice subjected to TS. Similarly, the percentage of cells coexpressing FG and either TrkA or CGRP was significantly reduced in the TS group (Fig. 3c, e and Fig. S4b). These findings indicate that mechanical unloading does not influence the number of sensory neurons in the DRGs but does impair sensory innervation inside the bone, resulting in reduced FG uptake and transport.

### Mechanical reloading restores both bone mass and sensory innervation

**Fig. 4:**
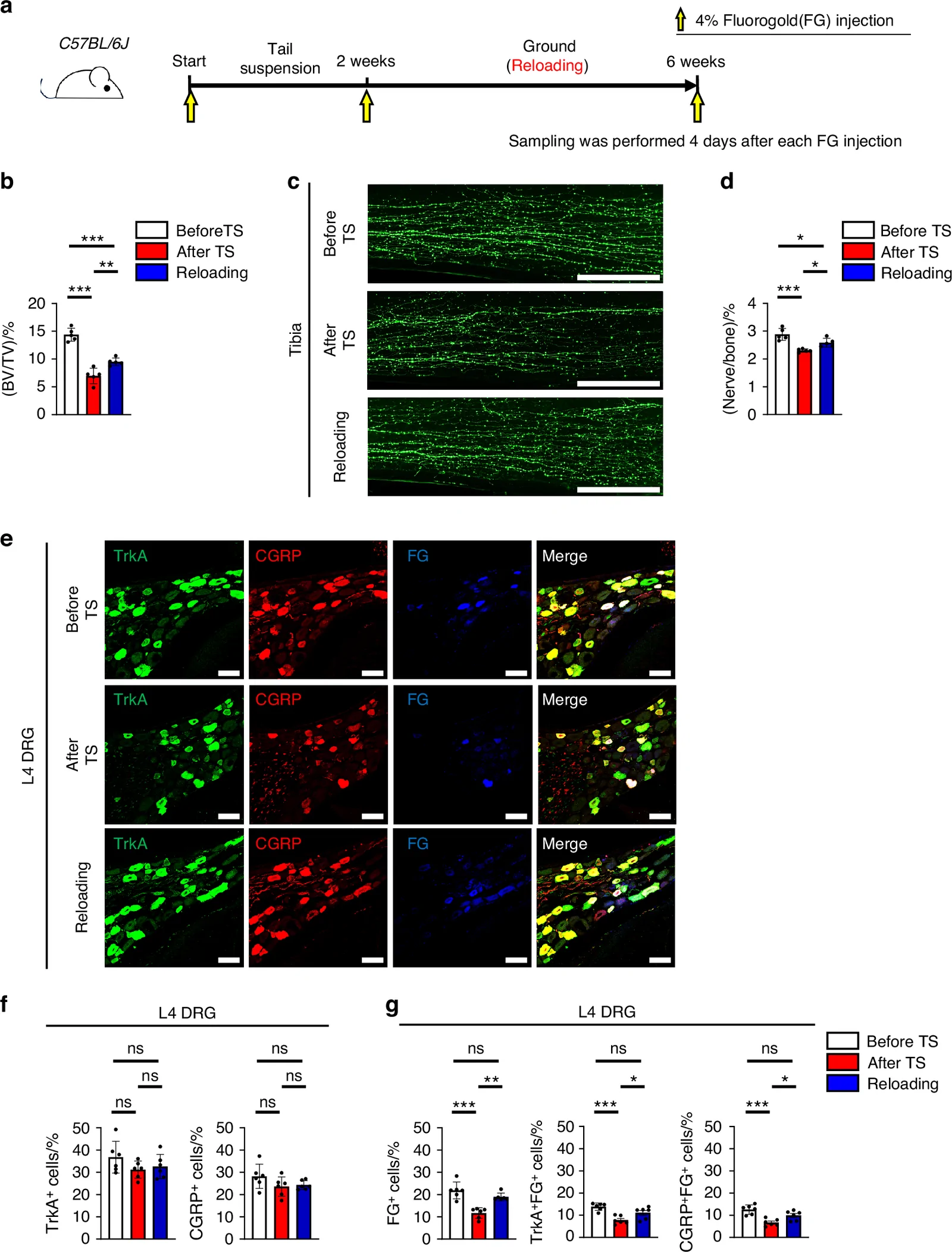
Mechanical reloading restores both bone mass and sensory innervation. **a** Experimental scheme of retrograde tracing at three time points: before TS, after 2 weeks of TS, and after 4 weeks of reloading following 2 weeks of TS. **b** Quantitative analysis of BV/TV of the proximal tibia at each time point (*n* = 5 per group). **c** Representative microscopy images of nerves inside the tibiae at each time point using the Osteo-DISCO method. Scale bar, 1 mm. **d** Quantitative analysis of the nerve/bone ratio(%) in the tibiae at each time point (*n* = 5 per group). **e** Representative IF images of the L4 DRG at each time point. Scale bar, 50 µm. **f** Quantitative analysis of the percentage of TrkA-, CGRP-, and FG-positive cells in the L4 DRG at each time point (*n* = 2; 6 sections per group). **g** Quantitative analysis of the percentages of FG-positive cells and cells coexpressing FG and either TrkA or CGRP in the L4 DRG at each time point (*n* = 2; 6 sections per group). The data are presented as the means ± SDs. One-way analysis of variance (ANOVA) with Tukey’s post hoc test was used for statistical analysis. *P < 0.05; **P < 0.01; ***P < 0.001; ns indicates not significant

Next, we investigated whether mechanical reloading could restore sensory innervation inside bone. To address this question, changes in sensory nerves inside the tibiae of male *C57BL/6J* mice were evaluated at three time points: before TS, after 2 weeks of TS, and after 4 weeks of reloading following 2 weeks of TS (Fig. 4a). Micro-CT analysis revealed that trabecular bone mass in the proximal tibiae decreased after 2 weeks of TS but partially recovered after 4 weeks of reloading (Fig. 4b). To evaluate the amount of nerve fibers inside the tibiae, age-matched *Sox10-Venus* mice were analyzed at the same three time points, and a partial recovery of nerve abundance by reloading was observed (Fig. 4c, d).

For retrograde tracing, 2 µL of 4% FG was injected into the proximal tibia of male *C57BL/6J* mice at each time point, and DRG samples were collected 4 days post-injection (Fig. 4a). Immunohistochemical analysis of the L3 and L4 DRGs revealed that while the percentages of TrkA-positive and CGRP-positive cells remained unchanged across all time points (Fig. 4e, f and Fig. S5a), the percentage of FG-positive cells, including those coexpressing TrkA or CGRP, significantly decreased after 2 weeks of TS and recovered after 4 weeks of reloading (Fig. 4e, g and Fig. S5b). These results demonstrate that sensory innervation inside bone is dynamically altered by mechanical unloading and reloading, in parallel with changes in trabecular bone mass.

### CGRP administration prevents bone loss induced by TS

**Fig. 5:**
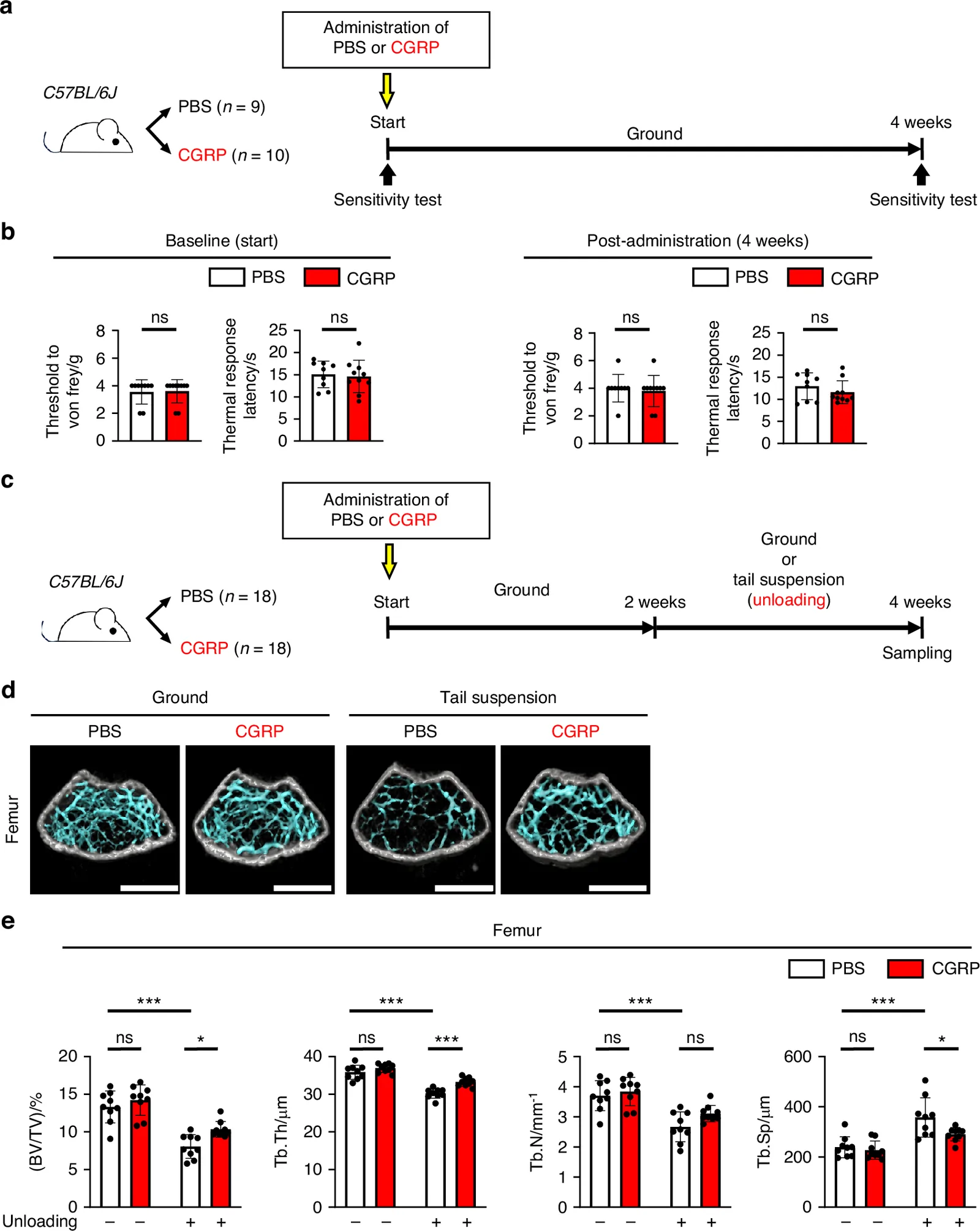
CGRP administration prevents bone loss induced by TS. **a** Schematic of the sensory tests performed on *C57BL/6J* mice continuously administered CGRP (*n* = 10) or PBS (*n* = 9) subcutaneously. **b** Sensory evaluation at baseline and after the administration of CGRP or PBS. Mechanical allodynia was assessed by the von Frey filament threshold in grams, and heat sensitivity was measured by the thermal response latency at 55 °C in seconds. **c** Schematic of the TS experiment involving *C57BL/6J* mice continuously administered CGRP (*n* = 9) or PBS (*n* = 9) subcutaneously. **d** Representative micro-CT images of the distal femurs of CGRP- or PBS-treated mice after 4 weeks of ground conditions and after 2 weeks of ground conditions followed by TS. Scale bar, 1 mm. **e** Quantitative analyses of bone volume and microstructural parameters of trabecular bone in the distal femur (*n* = 9 per group). The data are presented as the means ± SDs. Two-way analysis of variance (ANOVA) with Tukey’s post hoc test was used for statistical analysis. *P < 0.05; **P < 0.01; ***P < 0.001; ns indicates not significant

Our recent work demonstrated that CGRP-positive sensory nerves are crucial for maintaining bone homeostasis.^16^ Since mechanical unloading reduces the innervation of CGRP-positive sensory nerves inside bone (Fig. 3e and Fig. S4b), we hypothesized that CGRP administration might prevent unloading-induced bone loss. To verify this hypothesis, we continuously administered CGRP to 6-week-old male *C57BL/6J* mice for 4 weeks via an osmotic pump at a concentration of 2.4 µg/kg, a dose selected on the basis of previous studies,^16^ while control mice received PBS following the same protocol (Fig. 5a). Given the potential of CGRP to induce hypersensitivity, we first evaluated whether it alters pain sensitivity at the selected dose. Mechanical allodynia was assessed by applying von Frey filaments to the plantar region of the hind paws, and thermal sensitivity was evaluated by applying a hot plate (55 °C) on the plantar region of the hind paws. No significant differences were observed in the mechanical withdrawal threshold or thermal response latency between CGRP-treated and PBS-treated mice after 4 weeks of treatment (Fig. 5b). Next, to evaluate whether CGRP administration could prevent TS-induced trabecular bone loss, TS was initiated 2 weeks after osmotic pump implantation to avoid imposing dual stressors simultaneously (Fig. 5c). Micro-CT analysis revealed a significant decrease in trabecular bone mass in PBS-treated mice subjected to TS, and this decrease was attenuated in CGRP-treated mice (Fig. 5d, e). Such a protective effect was not observed in cortical bone (Fig. S6a). Quantitative PCR (qPCR) analyses revealed that TS decreased the expression levels of the osteogenic genes *Alpl* and *Ocn* in PBS-treated mice but not in CGRP-treated mice, whereas the expression levels of the osteoclastogenic genes *Acp5* and *Nfatc1* remained unchanged (Fig. S6b).

To address whether CGRP administration is also effective in skeletally mature mice, we repeated the same experiment using 10-week-old male *C57BL/6J* mice. Consistent with the results in 6-week-old mice, CGRP treatment showed the same trend of attenuating TS-induced trabecular bone loss (Fig. S6c). Furthermore, to assess the necessity of CGRP pretreatment prior to TS, we also conducted another experiment in which CGRP administration and TS were initiated simultaneously in 8-week-old male *C57BL/6J* mice. These results were similar to those of previous experiments with CGRP pretreatment (Fig. S6d), and serum osteocalcin levels tended to increase in CGRP-treated mice subjected to TS compared with those in PBS-treated mice (Fig. S6e).

These findings indicate that CGRP administration prevents unloading-induced bone loss by maintaining osteoblastic activity.

### Surgical nerve ablation inhibits loading-induced recovery of bone mass

Since the functional activation of sensory nerves by CGRP maintains bone mass during unloading, we next investigated whether these sensory nerves are essential for the recovery of trabecular bone mass after mechanical unloading. To address this question, we used a surgical nerve ablation model in which the nerves at the entry site into the tibia were surgically ablated.^16^ Six-week-old male *C57BL/6J* mice were used, and the right and left tibiae of each mouse were subjected to a sham operation and nerve ablation, respectively. We confirmed that the trabecular bone mass of nerve-ablated tibiae decreased after 2 weeks of ground conditions (Fig. S7a, b).

**Fig. 6:**
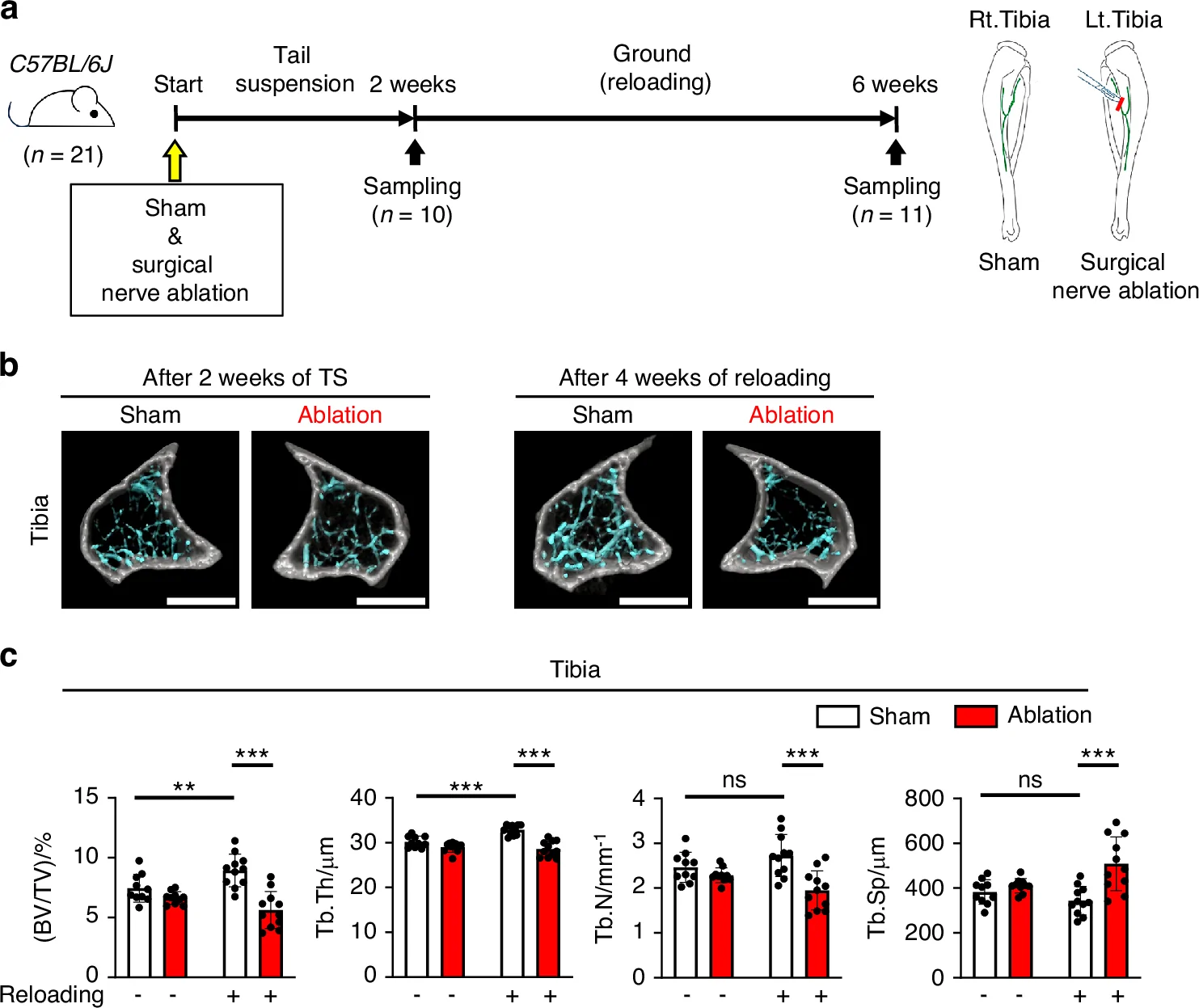
Surgical nerve ablation inhibits loading-induced recovery of trabecular bone mass. **a** Schematic of the experiment involving 2 weeks of TS followed by 4 weeks of mechanical reloading in *C57BL/6J* mice (*n* = 21) subjected to surgical nerve ablation. Before the beginning of the TS period, the right and left tibiae were subjected to a sham operation and nerve ablation, respectively. **b** Representative micro-CT images of sham-operated and nerve-ablated tibiae after 2 weeks of TS and after 4 weeks of mechanical reloading. Scale bar, 1 mm. **c** Quantitative analyses of bone volume and microstructural parameters in the proximal tibia after 2 weeks of TS (*n* = 10) and after 4 weeks of mechanical reloading (*n* = 11). The data are presented as the means ± SDs. Two-way mixed-effects model with Fisher’s LSD post hoc test was applied for statistical analysis. **P < 0.01; ***P < 0.001; ns indicates not significant

Using this model, mice were subjected to TS for 2 weeks, followed by mechanical reloading for 4 weeks (Fig. 6a). Micro-CT analysis revealed that the trabecular bone mass was comparable between nerve-ablated and sham-operated tibiae after 2 weeks of TS. However, after 4 weeks of mechanical reloading, only the sham-operated tibiae showed significant recovery of trabecular bone mass, whereas the nerve-ablated tibiae showed no improvement (Fig. 6b, c). qPCR analyses revealed that the expression levels of osteogenic genes increased after reloading in sham-operated tibiae but not in nerve-ablated tibiae (Fig. S7c). Furthermore, we repeated the same experiment using 10-week-old *C57BL/6J* mice to determine whether these findings are reproducible in skeletally mature mice. Consistent with the results in 6-week-old mice, trabecular bone mass recovery was specifically suppressed in nerve-ablated tibiae (Fig. S7d). These results indicate that nerves inside bone are essential for loading-induced bone recovery.

### Genetic ablation of sensory nerves also inhibits loading-induced recovery of bone mass

While surgical nerve ablation results in complete nerve elimination through the direct ablation of nerves entering the tibia, the use of this method for evaluating the specific effects of sensory nerves is limited since bones are innervated by both sensory and sympathetic nerves.^34,35^ Therefore, to further validate our findings using a less invasive approach, we performed genetic ablation of sensory nerves by crossing sensory nerve-specific Cre (*Advillin-Cre*) mice with diphtheria toxin receptor floxed (*DTR*^*fl/fl*^) mice to generate *Advillin-Cre;DTR floxed* (*Avil*^*Cre*^*;DTR*^*fl/+*^) mice.

IF staining revealed DTR protein only in DRGs harvested from *Avil*^*Cre+*^*;DTR*^*fl/+*^ mice. No difference in the expression of TrkA or CGRP was detected between *Avil*^*Cre+*^*;DTR*^*fl/+*^ and *Avil*^*Cre-*^*;DTR*^*fl/+*^ DRGs (Fig. S8a). PCR analysis also showed DTR expression predominantly in the lumbar DRG, with no expression detected in other tissues (Fig. S8b).

**Fig. 7:**
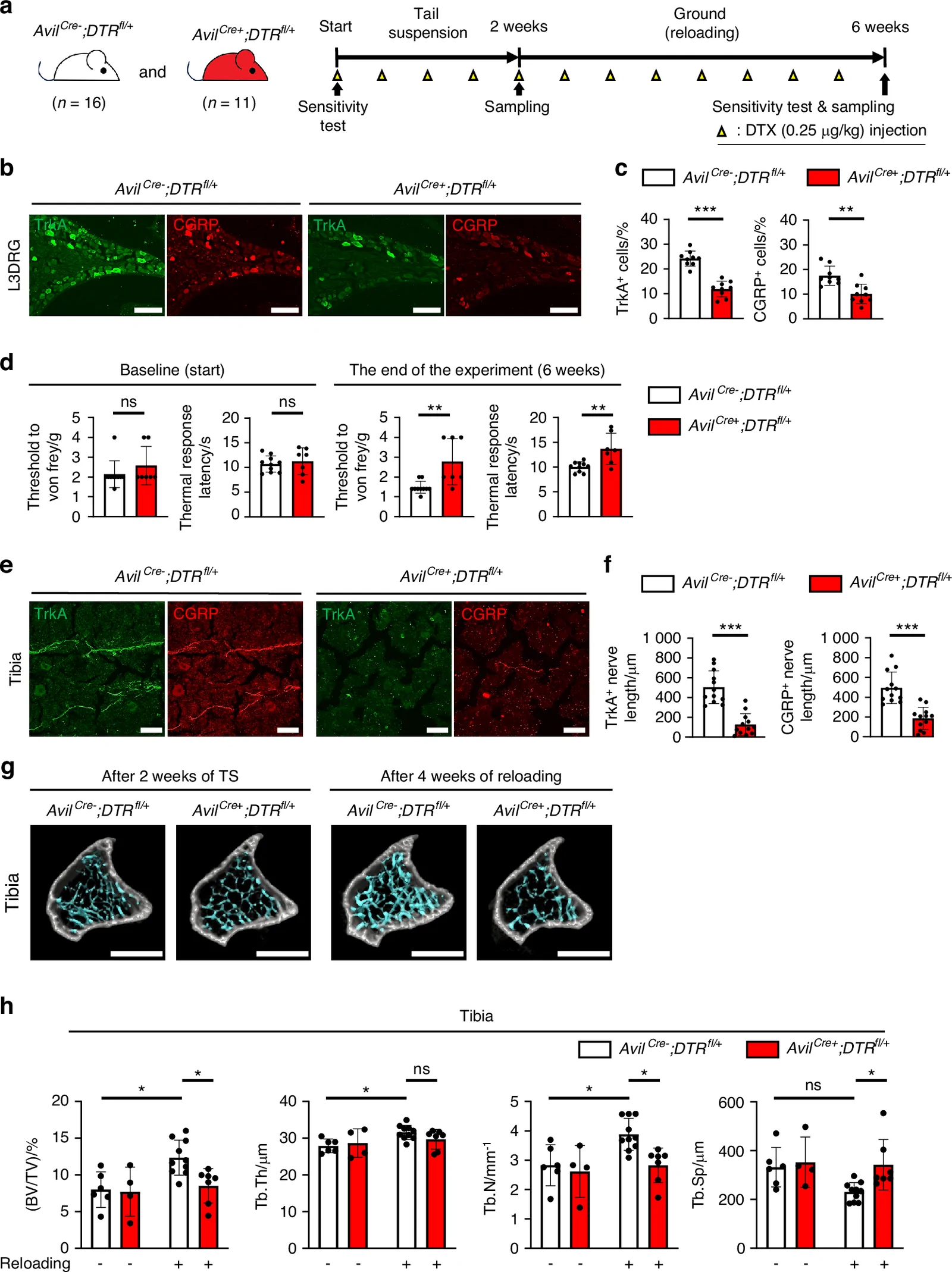
Genetic ablation of sensory nerves also inhibits loading-induced recovery of bone mass. **a** Schematic of the experiment involving 2 weeks of TS followed by 4 weeks of mechanical reloading on *Avil*^*Cre+*^*;DTR*^*fl/+*^ mice (*n* = 11) or *Avil*^*Cre-*^*;DTR*^*fl/+*^ mice (*n* = 16). DTX was intraperitoneally injected into all the mice twice a week throughout the experiment. **b** Representative IF images of the L3 DRG after mechanical reloading for 4 weeks. Scale bar, 100 µm. TrkA: tropomyosin receptor kinase A. CGRP: calcitonin gene-related peptide. **c** Quantitative analysis of the percentage of TrkA- and CGRP-positive cells in the L3 DRG (*n* = 3; 9 sections per group). **d** Sensory function of *Avil*^*Cre+*^*;DTR*^*fl/+*^ mice (*n* = 7) and *Avil*^*Cre-*^*;DTR*^*fl/+*^ mice (*n* = 10) at baseline and at the end of the experiment. Mechanical allodynia was assessed by the von Frey filament threshold in grams, and heat sensitivity was measured by the thermal response latency at 55 °C in seconds. **e** Representative IF images of TrkA- and CGRP-positive nerve fibers in the tibiae of *Avil*^*Cre+*^*;DTR*^*fl/+*^ and *Avil*^*Cre-*^*;DTR*^*fl/+*^ mice at the end of the experiment. Scale bar, 50 µm. **f** Quantitative analysis of the TrkA- and CGRP-positive nerve length in the tibia (*n* = 2; 12 sections per group). **g** Representative micro-CT images of the proximal tibiae of *Avil*^*Cre+*^*;DTR*^*fl/+*^ and *Avil*^*Cre-*^*;DTR*^*fl/+*^ mice after 2 weeks of TS and 4 weeks of mechanical reloading. Scale bar, 1 mm. **h** Quantitative analyses of the bone volume and microstructural parameters of the proximal tibiae of *Avil*^*Cre+*^*;DTR*^*fl/+*^ and *Avil*^*Cre-*^*;DTR*^*fl/+*^ mice after 2 weeks of TS and after 4 weeks of mechanical reloading. The data are presented as the means ± SDs. Two-tailed unpaired Student’s *t* test and two-way analysis of variance (ANOVA) with Tukey’s post hoc test were used for statistical analysis. *P < 0.05; **P < 0.01; ***P < 0.001; ns indicates not significant

Using this mouse model, 8-week-old male *Avil*^*Cre+*^*;DTR*^*fl/+*^ and *Avil*^*Cre-*^*;DTR*^*fl/+*^ mice were subjected to TS for 2 weeks followed by mechanical reloading for 4 weeks to evaluate the role of sensory nerves in the loading-induced recovery of trabecular bone mass. In this study, 0.25 µg/kg DTX was injected twice a week to ablate sensory nerves (Fig. 7a). At the end of the experiment, the percentages of TrkA-positive and CGRP-positive cells in the DRG were decreased in the *Avil*^*Cre+*^*;DTR*^*fl/+*^ mice (Fig. 7b, c). Additionally, in the behavioral tests, *Avil*^*Cre+*^*;DTR*^*fl/+*^ mice presented a significant increase in the von Frey filament threshold and a significantly delayed response in the hot plate test at the end of the experiment (Fig. 7d). Furthermore, we performed IF staining to measure the amount of sensory nerves inside the tibia and femur using anti-TrkA and anti-CGRP antibodies. IF staining revealed significantly fewer TrkA-positive and CGRP-positive sensory nerve fibers in the tibiae and femurs of the *Avil*^*Cre+*^*;DTR*^*fl/+*^ mice than in those of the *Avil*^*Cre-*^*;DTR*^*fl/+*^ mice (Fig. 7e, f and Fig. S9c, d). However, the expression of tyrosine hydroxylase (TH), which is a marker of adrenergic sympathetic nerves, did not differ (Fig. S9a–d). These results confirmed that sensory nerves were selectively ablated in the *Avil*^*Cre+*^*;DTR*^*fl/+*^ mice.

Micro-CT analysis of tibiae and femurs revealed no difference in the trabecular bone mass after 2 weeks of TS in either group. However, after 4 weeks of mechanical reloading, significant bone recovery was observed in the *Avil*^*Cre-*^*;DTR*^*fl/+*^ mice, whereas loading-induced recovery of trabecular bone mass was completely inhibited in the *Avil*^*Cre+*^*;DTR*^*fl/+*^ mice (Fig. 7g, h and Fig. S10c, d). Such recovery was not observed in cortical bone (Fig. S10e, f). qPCR analysis revealed that the expression levels of osteogenic genes increased after reloading in *Avil*^*Cre-*^*;DTR*^*fl/+*^ mice but not in *Avil*^*Cre+*^*;DTR*^*fl/+*^ mice, whereas the expression levels of osteoclastogenic genes remained unchanged (Fig. S11a). Consistent with these findings, bone histomorphometry revealed an increased osteoblast number (N.Ob/BS) and bone formation rate (BFR/BS) in *Avil*^*Cre-*^*;DTR*^*fl/+*^ mice, with no difference in the osteoclast number (N.Oc/BS) after reloading (Fig. S11b). Serum osteocalcin levels also tended to increase in *Avil*^*Cre-*^*;DTR*^*fl/+*^ mice (Fig. S11c). These findings suggest that sensory nerves play a critical role in loading-induced bone recovery by promoting osteoblastic activity.

The results obtained using these two approaches, surgical and genetic ablation of sensory nerves, indicate that sensory nerves inside bone are essential for maintaining bone homeostasis in response to changes in mechanical loading, primarily through the regulation of osteoblast-mediated bone formation.

## Discussion

Osteoporosis and fragility fractures have emerged as critical public health concerns in our aging society.^1^ Exposure to microgravity and reduced physical activity accelerate the development of musculoskeletal functional disorders.^2^ Although mechanical loading is essential for maintaining bone homeostasis, the precise mechanisms underlying this process remain poorly understood. Understanding these mechanisms is crucial for the development of novel therapeutic strategies to address age-related musculoskeletal disorders and increase healthy life expectancy.

Bone is a dynamic and adaptable tissue that undergoes continuous remodeling to maintain its strength.^36^ Recent research has shown that communication between bone cells and sensory nerves plays a key role in regulating bone formation in response to mechanical loading.^37^ Thomlinson et al. reported that mechanical loading causes osteoblasts to release NGF and induce sprouting of TrkA sensory nerves,^19^ and Gao et al. demonstrated that mechanical loading induces osteoblast-derived PGE2 expression and regulates bone formation through the sensory nerve‒hypothalamus‒sympathetic axis.^14^ Furthermore, Mei et al. reported that mechanical loading activates sensory nerves in the jaw and that the neurotransmitters released by these sensory nerves promote bone formation.^33^ While these studies have provided valuable insights into the mechanisms of bone–nerve communication during mechanical loading, the interaction between bone cells and sensory nerves during mechanical unloading remains unclear.

To address this topic, we employed three mechanical unloading models, namely, the TS, HLI, and spaceflight models.^38^ While ground-based experimental models, i.e., the TS and HLI models, are widely used to simulate mechanical unloading, they require the use of physical restraints and cannot fully recapitulate mechanical unloading under physiological conditions. In contrast, spaceflight experiments allow complete mechanical unloading without physical constraints, and space environments are known to promote aging-like processes in the musculoskeletal system.^20–24^ Therefore, to investigate bone–nerve communication during mechanical unloading, we designed spaceflight experiments in collaboration with JAXA. To accurately assess the effects of mechanical unloading on the musculoskeletal system, the mice were dissected, and tissue samples were collected in orbit to prevent the mice from being exposed to the effects of gravity upon their return to Earth. Furthermore, tissue fixation was conducted in orbit; specifically, 4% PFA was used to preserve Venus fluorescence signals for the identification of nerves. The tissue samples were immersed in 4% PFA for 16–24 h and then frozen and stored until a series of analyses were performed on Earth. This approach enabled us to evaluate neural changes in the musculoskeletal system under microgravity conditions and revealed a marked decrease in the amount of nerve fibers inside the bones of mice subjected to spaceflight (Fig. 1d, e). Importantly, these findings were reproduced in the other mechanical unloading models, i.e., the TS and HLI models (Fig. 2c, d, g, h). Although the TS and HLI models have limitations, this consistency across different experimental models strongly suggests that mechanical unloading is the primary driver of reduced nerve abundance inside bone.

We also investigated the nerve distribution in muscles such as the soleus and plantaris, which showed significant muscle weight loss in the spaceflight experiment (Fig. S2d). In contrast to the neural changes observed in bone, there was no change in the amount of nerve fibers in muscle (data not shown). This difference in response may be attributed to the different characteristics of bone and muscle tissues; for example, muscles are innervated by both sensory and motor nerves.^39^

Analysis of the vascular system revealed that EMCN- and VE-cadherin-positive vessels in bone tissue were well preserved and that the volume of these vessels remained unchanged after 3 weeks of spaceflight (Fig. S2e, f). Stabley et al. also demonstrated limited changes in vascular structure in skeletal muscles after 15 days in microgravity, with no significant alterations in vessel wall thickness or maximal intraluminal diameter.^40^ Together with our observations of vascular networks in bone, these findings suggest that the vascular structure in both bone and muscle tissues is well preserved after short-term microgravity exposure. Given that neural changes precede vascular changes during tissue repair and development,^18,41^ the nervous system is more sensitive than the vascular system to changes in gravity.

We performed retrograde tracing experiments to investigate whether the decrease in nerve abundance inside bone resulted from a reduction in the number of nerve cells or a decrease in innervation. Our analysis revealed that the percentage of TrkA-positive and CGRP-positive cells in the DRG remained unchanged, suggesting that the number of sensory neurons in the DRG is not affected by mechanical unloading (Fig. 3d and Fig. S4a). However, the percentage of FG-positive cells and cells coexpressing FG and either TrkA or CGRP was significantly reduced under mechanical unloading conditions (Fig. 3e and Fig. S4b). These findings indicate that mechanical unloading specifically impairs sensory innervation and the uptake and transport of the FG tracer inside bone. Conversely, mechanical reloading partially restored the nerve abundance inside bone (Fig. 4d) and increased the percentage of FG-positive cells coexpressing TrkA or CGRP in the DRG (Fig. 4g and Fig. S5b), indicating that sensory innervation dynamically responds to mechanical alteration in parallel with changes in trabecular bone mass. Most nerves inside bone are unmyelinated nerve fibers,^42,43^ which are known for their high plasticity.^44,45^ On the basis of this anatomical feature and previous studies demonstrating the potential of sensory nerves to adapt to mechanical loading,^46^ the changes in nerve abundance we observed likely reflect the dynamic plasticity of sensory nerve fibers rather than irreversible nerve degeneration.

CGRP administration during mechanical unloading significantly prevented trabecular bone loss through the regulation of osteoblastic activity (Fig. 5e and Fig. S6b). CGRP is known to upregulate osteogenic genes such as *Alpl* and *Ocn*^16,47,48^ and promote bone tissue repair.^49,50^ Since CGRP is released primarily from sensory nerve terminals,^51,52^ the reduction in sensory innervation by mechanical unloading likely leads to a decrease in CGRP levels in the bone cavity. Importantly, the results of the current study demonstrate that the trabecular bone mass was maintained in CGRP-treated mice (Fig. 5e and Fig. S6c, d). Thus, these results indicate that CGRP secretion from sensory nerve terminals is important for regulating trabecular bone mass.

Furthermore, this study revealed that surgical and genetic nerve ablation inhibited the recovery of trabecular bone mass in response to mechanical loading (Figs. 6c and 7h). While surgical nerve ablation can eliminate only nerves entering the tibia without affecting the surrounding muscles or other organs,^16^ it may eliminate both sensory and sympathetic nerves, limiting its use. To address this limitation, we generated *Avil*^*Cre*^*;DTR*^*fl/+*^ mice to achieve the selective ablation of sensory nerves. IF staining of bone tissue confirmed that only sensory nerves were targeted by this genetic approach, as evidenced by the preservation of the sympathetic nerve marker TH (Fig. S9b, d). At the cellular level, the results of the qPCR analyses and bone histomorphometry analyses revealed reduced osteoblastogenesis after reloading in both ablation models, whereas osteoclastogenesis remained unchanged (Figs. S7c, S11a, b). The results from these two independent approaches strongly indicate that sensory nerves play a crucial role in osteoblast-mediated bone formation.

We also confirmed that surgical or genetic nerve-ablated tibiae presented a decrease in trabecular bone mass after 2 weeks of ground conditions (Figs. S7a, b, S10a, b). However, although both nerve ablation models showed reduced trabecular bone mass after 4 weeks of reloading, no difference was observed after 2 weeks of TS before reloading (Figs. 6c and 7h). This is likely because TS-induced bone loss was dominant and masked the influence of nerve ablation.

**Fig. 8:**
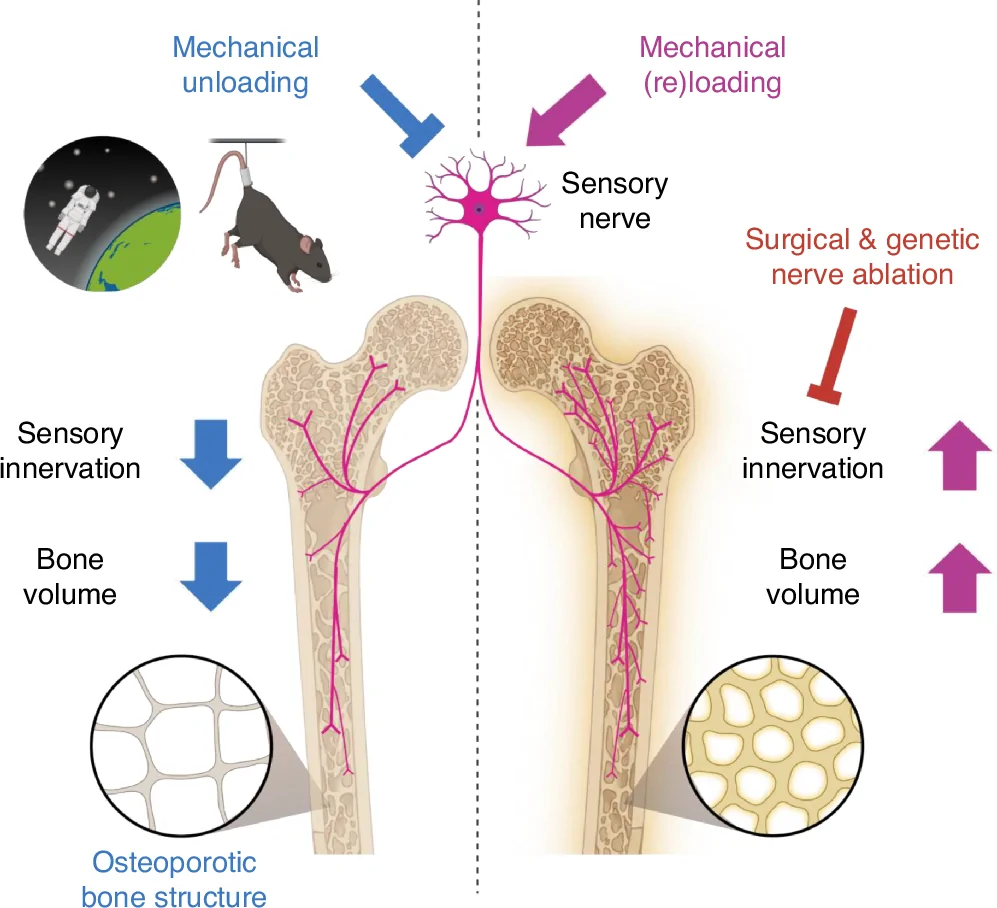
Sensory nerves inside bone respond to mechanical changes and regulate bone volume. A schematic summarizing the concept in the current study (Created with BioRender.com): Sensory nerve fibers originating from the DRG innervate bone tissue. Mechanical unloading, such as by TS or spaceflight, reduces sensory innervation inside bone and leads to trabecular bone loss, resulting in osteoporotic changes. In contrast, mechanical loading restores sensory innervation and trabecular bone mass, which is cancelled by the surgical or genetic ablation of sensory nerves. Thus, sensory nerves inside bone play a critical role in regulating bone homeostasis in response to mechanical changes

In this study, using spaceflight and ground-based experiments, we demonstrate that mechanical unloading induces trabecular bone loss and decreases sensory innervation inside bone. In contrast, mechanical reloading restores sensory innervation and trabecular bone mass. In addition, through experiments involving CGRP administration and surgical and genetic sensory nerve ablation, we found that sensory nerves inside bone orchestrate bone remodeling in response to mechanical unloading or reloading (Fig. 8). Although there are still questions regarding how mechanical unloading alters the detailed structure of sensory nerves inside bone, our findings that sensory nerves inside bone regulate bone homeostasis in response to mechanical loading may lead to the development of novel treatment strategies for bone loss induced by mechanical unloading, such as prolonged bed rest and long-term space travel.

## Materials and methods

### Mice

*C57BL/6J* mice, *Advillin-Cre* (*Avil-Cre*) mice, and DTR floxed (*DTR*^*fl/fl*^) mice were purchased from the Jackson Laboratory. *Sox10-Venus* transgenic mice were kindly provided by Dr. Chihiro Akazawa (Juntendo University School of Medicine, Tokyo, Japan). To generate *Advillin-Cre;DTR floxed* (*Avil*^*Cre*^*;DTR*^*fl/+*^) mice for functional studies, *Avil-Cre* male mice were crossed with *DTR*^*fl/fl*^ female mice. All the experiments were performed using male mice. All animal experiments were conducted with the approval of the Animal Study Committee of the Institute of Science Tokyo (approval number: #A2024-119C) and conformed to relevant institutional guidelines and regulations.

### Animal experiments

Spaceflight experiment: Fig. 1a shows an overview of the Mouse Habitat Unit 7 (MHU-7) mission. Six 11-week-old male *Sox10-Venus* mice were housed individually in a Transportation Cage Unit (TCU) and launched aboard SpaceX-24 from the NASA Kennedy Space Center (KSC) in Florida, USA, to the International Space Station (ISS) on December 21, 2021. Once on the ISS, each mouse was transferred into an individual Habitat Cage Unit (HCU) version 2 (HCU ver. 2, modified with A1600 water nozzles of Edstrom Industries Inc.). All the mice in HCUs were housed under microgravity in a JAXA Kibo centrifuge hardware Cell Biology Experimental Facility-Left (CBEF-L). After 3 weeks, the mice were transferred into the NASA Life Sciences Glovebox (LSG), and ketamine (150 mg/kg) and xylazine (45 mg/kg) were administered to the mice intraperitoneally. Then, the mice were euthanized by cardiac puncture followed by cervical dislocation. The right limbs were collected and immersed in 4% paraformaldehyde (PFA) for 16–24 h before being frozen, and the other whole bodies were frozen without PFA fixation. Unfortunately, one right limb had inadequate fixation because of insufficient immersion in 4% PFA. Therefore, all bone-related analyses, such as micro-CT analyses and quantitative analyses of nerve fibers or vascular networks inside bone, were conducted using the remaining five samples. The control group, which consisted of 6 age-matched male *Sox10-Venus* mice, was maintained in an ISSES (International Space Station Environment Simulator) chamber at KSC. The ISSES chamber replicated the flight habitat environment (temperature, humidity and CO_2_ levels). All animal experiments were conducted with the approval of the Animal Study Committee of Institute of Science Tokyo (Approval number: #A2024-119A), JAXA (Protocol number: 020-015D), and NASA (Protocol number: FLT-20-131, JAXA MHU-7) and in accordance with the guidelines and applicable laws in Japan and the United States of America.

Ground-based experiments for simulating microgravity: Eleven-week-old male *Sox10-Venus* mice were subjected to TS and HLI. Both procedures were performed as previously described.^53,54^ To validate the above spaceflight findings under terrestrial conditions, we used age-matched 11-week-old Sox10-Venus mice for the TS and HLI experiments.

Retrograde labeling of afferents in tibiae: six-week-old male *C57BL/6J* mice were assigned to either the ground or TS group. A total of 2 µL of 4% FG was injected into the right proximal tibia using a Hamilton syringe. Bone wax was applied over the injection site to prevent tracer leakage into the surrounding tissues. Four days after FG injection, the mice were deeply anesthetized and transcardially perfused with a solution of 4% PFA in PBS, and L3 and L4 DRGs were collected for analysis.

CGRP administration: six- to ten-week-old male *C57BL/6J* mice were used, and an osmotic pump (ALZET^®^; DURECT Corporation, Cupertino, CA, USA) was implanted subcutaneously in the dorsal region under sterile conditions, following previously described procedures.^16^ The pump was filled with either CGRP (4163-V; Peptide Institute, Inc., Osaka, Japan) dissolved in PBS at a concentration of 2.4 µg/kg or PBS alone as a vehicle control.

Nerve ablation study: Surgical nerve ablation was performed on 6- or 10-week-old male *C57BL/6J* mice as previously described.^16^ For genetic nerve ablation, 8-week-old male *Avil*^*Cre*^*;DTR*^*fl/+*^ mice were used, and DTX (Fujifilm Wako Pure Chemical Corporation, Osaka, Japan) was administered intraperitoneally.

Trabecular bone mass at the distal femur and proximal tibia peaks at 6 weeks of age in *C57BL/6J* male mice and decreases gradually with age.^55,56^ To eliminate the influence of physiological bone loss with age, we initially used 6-week-old mice for the tracing, CGRP administration, and surgical nerve ablation studies. However, 6-week-old mice are considered relatively young and skeletally immature. Thus, we repeated the CGRP administration and surgical nerve ablation experiments using 10-week-old mice, which are more skeletally mature.

### Micro-CT analysis

X-ray micro-CT analysis of murine femurs and tibiae was performed using the ScanXmateL090 system (Comscantecno). The images were analyzed using TRI/FCS-BON (Ratoc System Engineering) with the following parameters: X-ray tube potential: 90 kVp; X-ray intensity: 48 µA; number of projections: 1 200; integration time: 5.0 f/sec; Shepp-Logan filter; 992 slices on each axis; and resolution: 11.360 µm on each axis.

### Bone tissue clearing and image capture

To visualize the nerves inside bone, the samples were cleared using the Osteo-DISCO protocol as previously described.^16^ The femurs and tibiae were positioned face-down in the imaging chambers and immersed in silicone oil with a refractive index of 1.539 for imaging. We used a widefield microscope equipped with computational clearing technology (THUNDER Imager 3D Cell Culture with a Leica DFC9000GT camera; Leica Microsystems, Wetzlar, Germany)^57^ to acquire images. Images of Sox10-positive nerves were captured with a 10× objective (HL PL FLUOTAR 10×/0.32 dry; Leica Microsystems) at a wavelength of 475 nm with a laser intensity set to 75% and an exposure time of 500 ms under fluorescence imaging mode (FIM) with 100% illumination intensity (IL-Fld: 6). Z-stack images were acquired across the full thickness of the bone. Regions of interest (ROIs) were defined as three tiles distal to the lesser trochanter for femurs and three tiles proximal to the tibial tuberosity for tibiae (Fig. S1b). Instant computational clearing (ICC) was applied to all Z-stack images using Leica’s THUNDER algorithm, which removes background and out-of-focus light while preserving signal intensity for quantitative analysis. The ICC-processed Z-stack images were then converted into single images using maximum intensity projection (Fig. S1b). Quantitative analysis of nerve fibers was performed using ImageJ software (National Institutes of Health, Bethesda, MD, USA). The % nerve/bone ratio was defined as the proportion of the area occupied by nerve fibers within the total bone tissue area excluding the cortical bone tissue area in the captured images (Fig. S1b). This metric represents the amount of nerve fibers inside bone, and a consistent threshold was applied across all the studies.

For three-dimensional (3D) imaging, a light sheet fluorescence microscope (UltraMicroscope Blaze; Miltenyi Biotec GmbH, Bergisch Gladbach, Germany) was used, and 3D reconstruction and image processing were performed using Imaris x64 software (Version 9.2.1; Bitplane AG, Zurich, Switzerland).

### Immunohistochemistry

For immunohistochemical analysis, bones were fixed with 4% PFA for 4 h. After being washed three times with PBS, the bones were incubated with decalcification buffer (0.5 mol/L EDTA, pH adjusted to 7.5) for 48 h. After decalcification, the samples were cryoprotected in PBS containing 2% polyvinylpyrrolidone (PVP) and 20% sucrose for 24 h at 4 °C. The samples were then embedded in 4% carboxymethyl cellulose (CMC), snap-frozen in liquid nitrogen, and sliced into 20-µm sections using a cryostat (CryoStar NX70; Thermo Fisher Scientific Inc., Waltham, MA, USA). The sections were incubated with the following primary antibodies for 16 h at 4 °C: anti-CGRP antibody (#T4238, BMA Biomedicals), anti-TrkA antibody (AF1056, R&D Systems), anti-TH antibody (#AB152, Sigma–Aldrich), anti-EMCN antibody (#sc-65495, Santa Cruz Biotechnology), and anti-VE-cadherin antibody (AF1002, R&D Systems). 4’,6-Diamidino-2-phenylindole (DAPI) was used to stain the nuclei. The sections were observed using a confocal microscope (A1R; Nikon, FV3000; Olympus and LSM980 with Airyscan2 Multiplex; Zeiss).

For DRG observation, the samples were sliced into 8-µm sections using a cryostat. The sections were incubated with the following primary antibodies for 16 h at 4 °C: anti-CGRP antibody (#T4238, BMA Biomedicals), anti-TrkA antibody (AF1056, R&D Systems), and anti-human DTR (HB-EGF) antibody (AF-259-NA, R&D Systems). DAPI was used to stain the nuclei. The sections were observed using a confocal microscope (FV3000; Olympus) and a fluorescence microscope (BZ-9000; Keyence).

### Sensory tests

Sensory tests were performed as previously described.^15^ Briefly, the mice were habituated to plexiglass boxes (9.5 × 21 × 25 cm) and placed on an elevated perforated plastic platform for at least 2 h before behavioral testing. Mechanical sensitivity was assessed by applying calibrated von Frey filaments (0.02–8 g) to the plantar surface of the hind paw; a positive response was defined as brisk paw withdrawal. Heat sensitivity was assessed using a hot plate apparatus (NISSIN, Japan) maintained at 55 °C. The latency to hind paw licking or jumping was recorded, with a 30s cutoff time to prevent tissue damage.

### Quantitative PCR analysis

Total RNA was extracted from murine bone using TRIzol reagent (Invitrogen), and reverse transcription was performed with a ReverTra Ace qPCR RT Kit (TOYOBO) according to the manufacturer’s instructions. We performed a quantitative analysis of gene expression using the QuantStudio 1 Real-Time PCR System (Invitrogen). *Gapdh* expression served as an internal control.

The following primers were used:

*Acp5* sense, 5’-TCATGTCTCTGGGGGACAATTT-3’ and *Acp5* antisense, 5’- TTCCAGCGCTTGGAGATCTTAG-3’; *Alpl* sense, 5′-ACACCTTGACTGTGGTTACTGCTGA-3′ and *Alpl* antisense, 5′-CCTTGTAGCCAGGCCCGTTA-3′; *Gapdh* sense, 5′-ACCCAGAAGACTGTGGATGG-3′ and *Gapdh* antisense, 5′-CACATTGGGGGTAGGAACAC-3′; *Nfatc1* sense, 5′-CCGTTGCTTCCAGAAAATAACA-3′ and *Nfatc1* antisense, 5′-TGTGGGATGTGAACTCGGAA-3′; and *Ocn* sense, 5’- TCTGACAAAGCCTTCATGTCCA-3’, and *Ocn* antisense, 5’- CGGTCTTCAAGCCATACTGGTC-3’.

### Histomorphometry

For bone histomorphometry, we injected *Advillin-Cre;DTR floxed* (*Avil*^*Cre*^*;DTR*^*fl/+*^) mice with calcein (25 mg/kg; Sigma) intraperitoneally 5 and 2 days before sacrifice. After murine tibiae were harvested, the tibiae were embedded in methyl methacrylate resin, and the resin blocks were sectioned at 5 μm. Undecalcified sections of the tibiae were subjected to Villanueva bone staining. All bone histomorphometric parameters were measured in the secondary spongiosa region. To exclude the primary spongiosa, the measurement region was 0.23–1.16 mm distal to the lowest point of the growth plate. Static and dynamic histomorphometric analyses were performed using the Histometry RT CAMERA (System Supply, Nagano, Japan) at ×400 magnification at Niigata Bone Science Institute (Kizaki 761, Kita-Ku, Niigata city, Niigata, Japan). The results are reported as parameters recommended by the American Society for Bone and Mineral Research.

### ELISA

Serum osteocalcin levels were measured using the Mouse Gla-Osteocalcin High Sensitive EIA Kit (Takara Bio, Shiga, Japan; Cat. No. MK127) according to the manufacturer’s instructions.

### Statistical analysis

All the statistical analyses were performed with GraphPad Prism 10, and the statistical data are presented as the means ± standard deviations (SDs). Unpaired Student’s *t* test was used for two-group comparisons. One-way or two-way analysis of variance (ANOVA) with Tukey’s post hoc test was used for multi-group comparisons. With respect to the surgical nerve ablation experiments, a paired *t*-test or a two-way mixed-effects model with Fisher’s LSD post hoc test was used for statistical analysis. *P* < 0.05 was considered statistically significant.

## Supplementary information

Supplementary figures

Supplementary Movie 1

Supplementary Movie 2

## Data Availability

The data generated or analyzed during this study are included in the manuscript. All the data that support the findings of this study are available from the corresponding author upon reasonable request.
