# Supplementary material for: Sensory nerves regulate bone homeostasis in response to mechanical unloading or reloading: evidence from a spaceflight experiment and ground-based mechanical unloading models
Source: Bone Res. 2026 Aug 28;14:83. doi: 10.1038/s41413-026-00563-z (PMC13522514; doi:10.1038/s41413-026-00563-z)
Supplement: Supplementary file 1 [file 41413_2026_563_MOESM1_ESM.docx]

**Sensory nerves regulate bone homeostasis in response to mechanical unloading or reloading: Evidence from a spaceflight experiment and ground-based mechanical unloading models**

**<Authors>**

Shohei Tsujino^1^*, Hiroki Ochi^2^*, Risa Okada^3^, Daisuke Kamimura^3^, Kurando Utagawa^1,4^, Takaei Shin^5^, Hironori Yamada^6,7^, Aiko Unno^1^, Satoko Sunamura^1^, Chihiro Akazawa^8^, Masafumi Muratani^9^, Dai Shiba^3^, Toshitaka Yoshii^1^, Shingo Sato^1,10,11^

*These authors contributed equally to this work.

1. Department of Orthopaedic Surgery, Institute of Science Tokyo, Bunkyo-ku, Tokyo 113-8510, Japan
2. Department of Rehabilitation for Motor Functions, Research Institute, National Rehabilitation Center for Persons with Disabilities, Tokorozawa, Saitama 359-8555, Japan
3. Space Environment Utilization Center, Human Spaceflight Technology Directorate, JAXA, Tsukuba, Ibaraki, 305-8505, Japan
4. Department of Orthopaedics, Vanderbilt University Medical Center, 2200 Children's Way, Nashville, TN, 37232-9565, USA
5. Department of Medical Chemistry, Graduate School of Medicine, Kyoto University, Kyoto, 606-8501, Japan
6. Department of General Medicine, Institute of Science Tokyo, Bunkyo-ku, Tokyo 113-8510, Japan
7. Kawasaki Municipal Tama Hospital, Tama-ku, Kanagawa 214-8525, Japan
8. Intractable Disease Research Center, Juntendo University Graduate School of Medicine, Hongo, Bunkyo-ku, Tokyo 113-8421, Japan
9. Department of Genome Biology, Transborder Medical Research Center, Institute of Medicine, University of Tsukuba, Tsukuba, Ibaraki, 305-8575, Japan
10. Center for Innovative Cancer Treatment, Institute of Science Tokyo, Bunkyo-ku, Tokyo 113-8519, Japan
11. To whom correspondence should be addressed. 1-5-45 Yushima, Bunkyo-ku, Tokyo 113-8519, Japan Phone: +81-3-5803-4865 E-mail: satoshin.phy2@tmd.ac.jp (Shingo Sato)

This file includes Supplementary Figures 1-11 with their respective legends and legends for Supplementary Movies 1 and 2.

**Supplementary Figure 1**

**Supplementary Figure 1.**

**S1a** Representative high-magnification confocal microscopy image of nerves inside the tibia of a *Sox10-Venus* mouse cleared via the Osteo-DISCO method. Nerve fibers are labeled by green fluorescence; Schwann cell nuclei (white arrowheads) show particularly strong fluorescent signals. Scale bars, 50 µm.

**S1b** (Upper) Schematic illustrations of the regions of interest (ROIs) used for nerve imaging and quantification. For the tibiae, three tiles were scanned proximally from the tibial tuberosity. For femurs, three tiles were scanned distally from the lesser trochanter. (Lower) Both nerve images represent nerves in the marrow cavity, and we measured the proportion of the area occupied by nerve fibers within the total bone tissue area excluding the cortical bone tissue area (asterisk) in the captured images. The dotted lines represent the border between the cortical bone and the marrow cavity.

**Supplementary Figure 2**

**Supplementary Figure 2. Microgravity exposure in space induces bone loss and decreases nerve abundance inside bone**

**S2a** Representative micro-CT images of distal femurs in the ground and spaceflight groups. Scale bar, 1 mm.

**S2b** Quantitative analyses of bone volume and microstructural indices of trabecular bone in the distal femur (n = 6 on the ground and n = 5 in space).

**S2c** Quantitative analyses of the geometric indices of cortical bone in the tibia and femur (n = 6 on the ground and n = 5 in space).

**S2d** Quantitative analyses of the weight of lower extremity muscles (n = 6 per group).

**S2e** Representative IF images of endomucin (EMCN)- and VE-cadherin-positive vessels inside the femur in the ground and spaceflight groups. Scale bars, 100 µm.

**S2f** Quantitative analysis of the percentages of EMCN- and VE-cadherin-positive areas (n = 6 on the ground and n = 5 in space).

The data are presented as the means ± SDs. Two-tailed unpaired Student’s t tests were used for statistical analysis. *P < 0.05; **P < 0.01; ***P < 0.001; ns indicates not significant.

**Supplementary Figure 3**

**Supplementary Figure 3. Quantitative analyses of cortical bone in the tail suspension (TS) and hindlimb immobilization (HLI) models**

**S3a, b** Quantitative analyses of the geometric indices of cortical bone in the tibiae (a) and femurs (b) of mice subjected to TS (n = 8 per group).

**S3c, d** Quantitative analyses of the geometric indices of cortical bone in the tibiae (c) and femurs (d) of mice subjected to HLI (n = 7 per group).

The data are presented as the means ± SDs. Two-tailed unpaired Student’s t tests were used for statistical analysis. *P < 0.05; **P < 0.01; ns indicates not significant.

**Supplementary Figure 4**

**Supplementary Figure 4.** **Mechanical unloading affects sensory innervation inside bone**

**S4a** Quantitative analysis of the percentages of TrkA- and CGRP-positive cells in the L3 DRG (n = 3; 9 sections per group).

**S4b** Quantitative analysis of the percentages of FG-positive cells and cells coexpressing FG and either TrkA or CGRP in the L3 DRG (n = 3; 9 sections per group).

The data are presented as the means ± SDs. Two-tailed unpaired Student’s t tests were used for statistical analysis. ***P < 0.001; ns indicates not significant.

**Supplementary Figure 5**

**Supplementary Figure 5. Mechanical reloading recovers sensory innervation inside bone**

**S5a** Quantitative analysis of the percentages of TrkA- and CGRP-positive cells in the L3 DRG (n = 2; 6 sections per group).

**S5b** Quantitative analysis of the percentages of FG-positive cells and cells coexpressing FG and either TrkA or CGRP in the L3 DRG (n = 2; 6 sections per group).

The data are presented as the means ± SDs. One-way analysis of variance (ANOVA) with Tukey’s post hoc test was used for statistical analysis. *P < 0.05; **P < 0.01; ***P < 0.001; ns indicates not significant.

**Supplementary Figure 6**

**Supplementary Figure 6. CGRP administration prevents bone loss induced by TS**

**S6a** Quantitative analyses of the geometric indices of cortical bone in the femurs of CGRP- or PBS-treated mice (n = 9 per group).

**S6b** Quantitative PCR analyses of osteogenic genes, including *Alpl* and *Ocn*, and osteoclastogenic genes, including *Acp5* and *Nfatc1,* in bone samples (n = 4 per group).

**S6c** Quantitative analyses of bone volume and microstructural parameters of trabecular bone in the distal femurs of 10-week-old *C57BL/6J* mice pretreated with CGRP (n = 6 per group).

**S6d** Quantitative analyses of bone volume and microstructural parameters of trabecular bone in the distal femurs of 8-week-old *C57BL/6J* mice without CGRP pretreatment (n = 6 per group).

**S6e** Serum osteocalcin levels measured by ELISA (n = 6 per group).

The data are presented as the means ± SDs. Two-way analysis of variance (ANOVA) with Tukey’s post hoc test was used for statistical analysis. *P < 0.05; **P < 0.01; ***P < 0.001; ns indicates not significant.

**Supplementary Figure 7**

**Supplementary Figure 7. Surgical nerve ablation inhibits loading-induced recovery of trabecular bone mass**

**S7a** Schematic of the experiment involving 2 weeks of ground conditions in *C57BL/6J* mice subjected to surgical nerve ablation. Before the beginning of the experiment, the right and left tibiae were subjected to a sham operation and nerve ablation, respectively.

**S7b** Quantitative analyses of bone volume/tissue volume (BV/TV) in the proximal tibia after 2 weeks of ground conditions (n = 3 per group).

**S7c** Quantitative PCR analyses of osteogenic genes, including *Alpl* and *Ocn*, and osteoclastogenic genes, including *Acp5* and *Nfatc1,* in bone samples (n = 4 per group).

**S7d** Quantitative analyses of bone volume and microstructural parameters in the proximal tibia of 10-week-old *C57BL/6J* mice after 2 weeks of TS and after 4 weeks of mechanical reloading (n = 6 per group).

The data are presented as the means ± SDs. A paired t-test or a two-way mixed-effects model with Fisher’s LSD post hoc test was used for statistical analysis. *P < 0.05; **P < 0.01; ***P < 0.001; ns indicates not significant.

**Supplementary Figure 8**

**Supplementary Figure 8. DTR is specifically expressed in sensory neurons in *Avil^Cre+^;DTR^fl/+^* mice**

**S8a** Representative IF images of the L3 DRG before DTX administration. Scale bar, 100 µm. DTR: diphtheria toxin receptor, TrkA: tropomyosin receptor kinase A.

**S8b** PCR analysis of DTR expression in various tissues from *Avil^Cre+^;DTR^fl/+^* mice, showing specific expression in the DRG.

**Supplementary Figure 9**

**Supplementary Figure 9. Sensory nerves are specifically ablated in *Avil^Cre+^;DTR^fl/+^* mice**

**S9a** Representative IF images of TH-positive nerve fibers in the tibiae of *Avil^Cre+^;DTR^fl/+^* and *Avil^Cre-^;DTR^fl/+^* mice at the end of the experiment. Scale bar, 50 µm.

**S9b** Quantitative analysis of the TH-positive nerve length in the femur (n = 2; 12 sections per group).

**S9c** Representative IF images of TrkA-, CGRP- and TH-positive nerve fibers in the femurs of *Avil^Cre+^;DTR^fl/+^* and *Avil^Cre-^;DTR^fl/+^* mice at the end of the experiment. Scale bar, 50 µm.

**S9d** Quantitative analysis of the TrkA-, CGRP- and TH-positive nerve length in the femur (n = 2; 12 sections per group).

The data are presented as the means ± SDs. Two-tailed unpaired Student’s t test was used for statistical analysis. ***P < 0.001; ns indicates not significant.

**Supplementary Figure 10**

**Supplementary Figure 10. Genetic sensory nerve ablation inhibits loading-induced trabecular bone recovery**

**S10a** Schematic of the experiment involving 2 weeks of ground conditions on *Avil^Cre+^;DTR^fl/+^* and *Avil^Cre-^;DTR^fl/+^* mice. DTX was intraperitoneally injected into all the mice twice a week throughout the experiment.

**S10b** Quantitative analysis of bone volume/tissue volume (BV/TV) of the proximal tibiae of the *Avil^Cre+^;DTR^fl/+^* and *Avil^Cre-^;DTR^fl/+^* mice after 2 weeks of ground conditions (n = 3 per group).

**S10c** Representative micro-CT images of the distal femurs of *Avil^Cre+^;DTR^fl/+^* and *Avil^Cre-^;DTR^fl/+^* mice after 2 weeks of TS and 4 weeks of mechanical reloading. Scale bar, 1 mm.

**S10d** Quantitative analyses of bone volume and microstructural parameters in the distal femurs of *Avil^Cre+^;DTR^fl/+^* and *Avil^Cre-^;DTR^fl/+^* mice after 2 weeks of TS and after 4 weeks of mechanical reloading.

**S10e, f** Quantitative analyses of the geometric indices of cortical bone in the tibiae (e) and femurs (f) of *Avil^Cre+^;DTR^fl/+^* and *Avil^Cre-^;DTR^fl/+^* mice after 2 weeks of TS and after 4 weeks of mechanical reloading.

The data are presented as the means ± SDs. Two-tailed unpaired Student’s t test and two-way analysis of variance (ANOVA) with Tukey’s post hoc test were used for statistical analysis. *P < 0.05; **P < 0.01; ns indicates not significant.

**Supplementary Figure 11**

**Supplementary Figure 11. Sensory nerve ablation affects osteoblast activity and bone formation**

**S11a** Quantitative PCR analysis of osteogenic genes, including *Alpl* and *Ocn*, and osteoclastogenic genes, including *Acp5* and *Nfatc1,* in bone samples from *Avil^Cre+^;DTR^fl/+^* and *Avil^Cre-^;DTR^fl/+^* mice (n = 4 per group).

**S11b** Osteoblast and osteoclast parameters assessed by bone histomorphometry in *Avil^Cre+^;DTR^fl/+^* and *Avil^Cre-^;DTR^fl/+^* mice. N.Ob/BS: osteoblast number/bone surface, N.Oc/BS: osteoclast number/bone surface, N.Ob/N.Oc ratio: ratio of N.Ob/BS to N.Oc/BS, BFR/BS: bone formation rate/bone surface (n = 4 per group).

**S11c** Serum osteocalcin levels measured by ELISA in *Avil^Cre+^;DTR^fl/+^* and *Avil^Cre-^;DTR^fl/+^* mice (n = 4 per group).

The data are presented as the means ± SDs. Two-way analysis of variance (ANOVA) with Tukey’s post hoc test was used for statistical analysis. *P < 0.05; **P < 0.01; ns indicates not significant.

**Supplementary Movie 1.**

Video showing a three-dimensional image of neural networks inside the tibia of a *Sox10-Venus* mouse captured by an UltraMicroscope Blaze light sheet microscope (Miltenyi Biotec). The green-labeled nerves enter the bone cavity through a specific site posterior to the tibial diaphysis.

**Supplementary Movie 2.**

Video showing a three-dimensional image of neural networks inside the femur of a *Sox10-Venus* mouse captured by an UltraMicroscope Blaze light sheet microscope (Miltenyi Biotec). The green-labeled nerves enter the bone from the trochanter and innervate the bone cavity.
